# Blood immune transcriptome analysis of artificially fed dairy calves and naturally suckled beef calves from birth to 7 days of age

**DOI:** 10.1038/s41598-018-33627-0

**Published:** 2018-10-18

**Authors:** C. Surlis, B. Earley, M. McGee, K. Keogh, P. Cormican, G. Blackshields, K. Tiernan, A. Dunn, S. Morrison, A. Arguello, S. M. Waters

**Affiliations:** 1 Teagasc Animal and Bioscience Research Department, Grange, Dunsany, Meath, Ireland; 2 Sustainable Livestock, Agri-food and Bio-sciences Institute, BT26 6DR Hillsborough, United Kingdom

## Abstract

Neonatal calves possess a very immature and naïve immune system and are reliant on the intake of maternal colostrum for passive transfer of immunoglobulins. Variation in colostrum management of beef and dairy calves is thought to affect early immune development. Therefore, the objective of this study was to examine changes in gene expression and investigate molecular pathways involved in the immune-competence development of neonatal Holstein dairy calves and naturally suckled beef calves using next generation RNA-sequencing during the first week of life. Jugular whole blood samples were collected from Holstein (H) dairy calves (n = 8) artificially fed 5% B.W. colostrum, and from beef calves which were the progenies of Charolais-Limousin (CL; n = 7) and Limousin-Friesian beef suckler cows (LF; n = 7), for subsequent RNA isolation. In dairy calves, there was a surge in pro-inflammatory cytokine gene expression possibly due to the stress of separation from the dam. LF calves exhibited early signs of humoral immune development with observed increases in the expression genes coding for Ig receptors, which was not evident in the other breeds by 7 days of age. Immune and health related DEGs identified as upregulated in beef calves are prospective contender genes for the classification of biomarkers for immune-competence development, and will contribute towards a greater understanding of the development of an immune response in neonatal calves.

## Introduction

New-born calves are immunologically naïve at birth, offering the ideal scenario to observe the development of immunocompetence through time. The protection of the womb environment during the pre-partum period, coupled with syndesmochorial placentation, results in a lack of experience to pathogens, meaning that calves are born with an essentially non-functional immune response^1^. Development of immunocompetence in calves relies on successful absorption of maternal colostrum derived immunoglobulins, which in turn is dependent upon successful colostrum management. The importance of colostrum-derived passive immunity, through intestinal absorption of colostral immunoglobulins, to the mortality, morbidity, and subsequent growth and welfare of a newborn calf is recognised internationally^2–5^. Consequently, colostrum feeding management is of critical importance to the health and vitality of the calf, in order to confer protection from the various septicemic and enteric diseases that they are susceptible to in early life^2^. Failure of passive transfer of colostrum derived IgGs (FPT, serum IgG < 10 mg/mL) markedly increases morbidity and mortality in calves^3–5^. There is tremendous variation in the passive immune status of dairy calves^3,6–8^ and, generally passive immunity of dairy calves is much lower than beef calves^7^. This difference is primarily attributed to differences in colostrum Ig levels, whereby dairy cows produce relatively large volumes of colostrum with relatively low concentrations of Ig while beef cows produce the opposite^3,9,10^. In the suckled beef calf, there are also large differences in passive immunity between cow breed types^3,8^.

The use of a systems approach such as RNA sequencing offers advantages over other molecular based techniques such as microarray, enabling unbiased opportunities towards the profiling of developing immunocompetence using a global unbiased view of relative transcriptomic alterations^11^. Peripheral whole blood samples are commonly used for immunological studies as they are easily obtained and may provide an insight into immune development, particularly when combined with a transcriptomics approach^12^. Previous studies from our group have successfully investigated the immune response of two dairy breeds to gradual weaning using whole blood to analyse alterations in the relative abundance of key immune genes^13^. Here, utilizing the whole blood transcriptome of dairy calves in addition to two beef breeds, we aim to elucidate the molecular mechanisms involved in the development of immunocompetence, from birth through the first 7 days of life. Understanding such mechanisms would be a step towards integrating optimum husbandry practices, and to identifying possible biomarkers associated with development of immunocompetence for breeding of superior calves.

## Results

### IgG concentration

Serum IgG concentrations at 0 h, 48 h, 72 h and 168 h post birth in dairy and beef calves are shown in Table 1. There was a significant effect of breed (P < 0.05) sampling time (P < 0.0001) and breed × sampling time interaction (P < 0.0001) for serum IgG concentrations. As expected, at 0 h, prior to the first feed of colostrum, baseline serum IgG concentrations were lower (P < 0.0001) compared with all other sampling times. In LF calves, serum IgG concentrations were greater (P < 0.001) compared to dairy calves at 48, 72 and 168 h post-birth and were not different from CL, except at 168 h when concentrations were lower in CL compared with LF. Colostrum IgG concentrations (mean (SD)) were not different across the three cow breed types (H; 63.1 (14.4), CL; 63.0 (25.2) LF (71.0 (14.5) mg/mL).

**Table 1:** Least square means (SEM) for serum IgG concentrations (mg/mL) from birth (d 0) to 168 h hours (h) for the calf progenies of H, CL and LF cows.

	0 h	12 h	24 h	48 h	72 h	168 h	SE	T	S	T × S
H	0.64^a^	14.34^b,x^	13.59^b,x^	14.47^b,x^	13.47^b,x^	9.01^b,x^	1.51	0.04	P < 0.0001	P < 0.0001
CL	0.01^a^	14.34^b^	18.34^b,x^	17.19^b^	15.93^b^	14.35^b,z^	1.61			
LF	0.01^a^	19.34^b,y^	24.91 ^b,y^	22.31^b,y^	19.57^b,y^	17.75^b,y^	1.61			

T; Treatment, S (sampling time); H = Holstein; CL = Charolais × Limousin (n = 7), LF = Limousin × Friesian (n = 7); ^a,b^Within rows, Lsmeans differ from d 0 by P < 0.05. ^x,y,z^Within columns Lsmeans differ across breed by P < 0.05.

### Differentially expressed genes

Overall relative gene expression levels in comparisons that yield significant levels of DEG are represented in Supplementary Figs 1–4 using principal component analysis and by mapping DEG using volcano plots. This plot represents overall relative gene expression abundances within each sample group and highlights similarities and differences in overall expression levels of transcripts in individual sample sets. Each comparison made in dairy and beef calves of levels of DEG present in each group are also presented as Venn diagrams in Supplementary Figs 5–9. Total numbers of DEG for each comparison are shown in Table 2. Lists of all DEG found in each comparisons are shown in Supplementary Tables 1–7. RNAseq data from the current study are available on NCBI’s Gene Expression Omnibus through GEO Series accession number GSE114432.

**Table 2:** Levels of upregulated and downregulated DEG across all comparisons and over-represented networks across comparisons with sufficient levels of DEG.

Comparison Group	Upregulated DEG	Downregulated DEG	Enriched Networks
**Dairy calves compared over time**			
Dairy 48 h versus Dairy 0 h	140	613	Post-Translational Modification, Small Molecule Biochemistry, Cellular Movement
			Immunological Disease, Infectious Diseases, Organismal Injury and Abnormalities
			Amino Acid Metabolism, Post-Translational Modification, Small Molecule Biochemistry
			Cell Death and Survival, Cellular Compromise, Inflammatory Response
			Cell Cycle, Cellular Development, Free Radical Scavenging
Dairy 168 h versus Dairy 72 h	572	1156	Cell Cycle, Cellular Assembly and Organization, DNA Replication, Recombination, and Repair
			Cell Cycle, Cellular Assembly and Organization, DNA Replication, Recombination, and Repair
			Cell Signalling, Molecular Transport, Vitamin and Mineral Metabolism
			Cellular Development, Cellular Growth and Proliferation, Endocrine System Disorders
			DNA Replication, Recombination, and Repair, Gene Expression, Cell Cycle
**Beef crossbreed calves compared at each time point**			
Beef CL 48 h versus Beef LF 48 h	539	177	Connective Tissue Disorders, Hereditary Disorder, Organismal Injury and Abnormalities
			Cell Death and Survival, Neurological Disease, Organismal Injury and Abnormalities
			Infectious Diseases, Cell-To-Cell Signalling and Interaction, Cellular Movement
			Cell Death and Survival, Connective Tissue Disorders, Hereditary Disorder
			Infectious Diseases, Antimicrobial Response, Inflammatory Response
Beef CL 72 h versus Beef LF 72 h	2	0	N/A
Beef CL 168 h versus Beef LF 168 h	0	0	N/A
**Beef LF calves compared over time**			
Beef LF 48 h versus Beef LF 0 h	1240	781	Cell Cycle, Cell Death and Survival, Cellular Assembly and Organization
			Cell Signalling, Molecular Transport, Vitamin and Mineral Metabolism
			Dermatological Diseases and Conditions, Developmental Disorder, Hereditary Disorder
			Lipid Metabolism, Small Molecule Biochemistry, Vitamin and Mineral Metabolism
			Carbohydrate Metabolism, Small Molecule Biochemistry, Embryonic Development
Beef LF 168 h versus Beef LF 72 h	535	1362	Cell-To-Cell Signalling and Interaction, Cell Signalling, Molecular Transport
			Cardiovascular Disease, Cell-To-Cell Signalling and Interaction, Inflammatory Response
			Carbohydrate Metabolism, Molecular Transport, Small Molecule Biochemistry
			Carbohydrate Metabolism, Endocrine System Disorders, Hereditary Disorder
			Cell-mediated Immune Response, Cellular Development, Cellular Function and Maintenance
**Beef CL calves over time**			
Beef CL 48 h versus Beef CL 0 h	1141	954	Cancer, Connective Tissue Disorders, Developmental Disorder
			Cell Cycle, Reproductive System Development and Function, Cell Death and Survival
			Cell-To-Cell Signalling and Interaction, Immunological Disease, Inflammatory Disease
			Connective Tissue Development and Function, Connective Tissue Disorders, Nervous System Development and Function
			Cardiovascular System Development and Function, Cell Cycle, Skeletal and Muscular System Development and Function
Beef CL 168 h versus Beef CL 72 h	45	338	Post translational modification, Cellular movement
			Cellular movement, Dermatological diseases, Cell-Cell signalling and interaction
			Cellular movement, Immune cell trafficking
			Developmental disorder, Hereditary disorder, Immunological disease
			Cell-Cell signalling and interaction, Haematological system development

### Pathway analysis across all comparisons

#### Over enriched networks in dairy calves over the first 168 h post birth

Comparisons of dairy calves at 0 h, 48 h, 72 h and 168 h, were examined for the presence of enriched molecular and cellular functional networks, physiological system developmental networks, and for the presence of biologically interesting canonical pathways, which are shown in Table 2.

**Figure 1:**
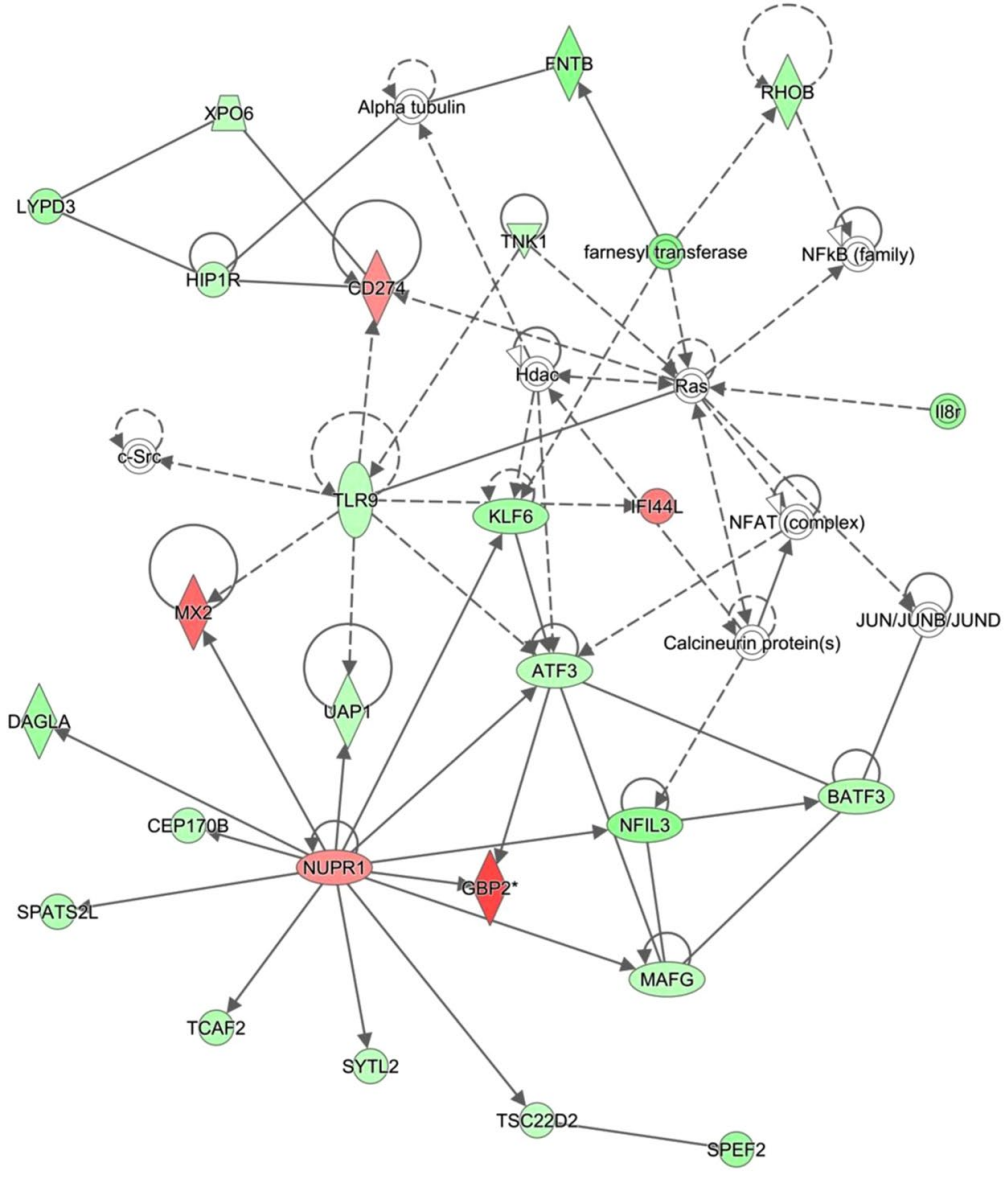
Immunological disease and Infectious disease network enriched in comparison of Dairy 48 h v Dairy 0 h. Network 2- Immunological Disease, Infectious Diseases, Organismal Injury and Abnormalities network enriched in comparison of dairy 48 h to 0 h. The network is displayed graphically as nodes (genes). The node colour intensity indicates the expression of genes; with red representing up-regulation and green, down-regulation in 48 h dairy calves compared to 0 h dairy calves.

The top enriched pathway in the comparison of 0 h calves to those sampled at 48 h was the role of pattern recognition receptor in recognition of bacteria and viruses, in which enriched genes were present at higher levels of abundance in the 0 h samples. The complement system pathway was also enriched in the comparison, with the majority of the genes present at higher levels in the 48 h calves than in the 0 h. Enriched networks included ‘Post translational modification’ (Network 1) and ‘Immunological disease’ (Network 2, Fig. 1).

Comparison of the transcriptomic profile of calves aged 168 h to those sampled at 72 h highlighted *1l-8* signalling as the most enriched canonical pathway, with the majority of genes present at lower levels in the 168 h calves than in the 72 h. Networks of interest included ‘Cell signalling, Molecular transport, Vitamin and mineral transport’ (Network 3), and ‘Cellular development, Cellular growth and Proliferation’ (Network 4). On investigation of any upstream effects of DEG enriched in the comparison of 168 h and 72 h dairy calves, we identified inhibition of a growth factor at 168 h, *TGFβ1*. Additionally, inhibition of a transmembrane receptor, *EPOR*, was identified as −5.487 times lower in the 168 h calves, with 22 out of the proposed 366 genes involved in its upstream regulation present in the DEG list.

#### Over enriched networks in beef calves over the first 168 h post birth

(i) Comparisons over time within crossbreeds: Comparisons were carried out within each beef breed across the four time points, 0 h, 48 h, 72 h and 168 h, to investigate the transition of the developing immune response over time. The top over-represented networks are highlighted in Table 2. Firstly, biologically interesting pathways were investigated within the CL crossbred calves. Comparison of 48 h to 0 h CL calves resulted in 2095 DEG, 1766 of which were successfully mapped in IPA. Biologically interesting enriched molecular, cellular and physiological networks included cellular movement, immune cell trafficking, and lymphoid tissue structure and development. Top enriched canonical pathways included cholesterol biosynthesis, which was upregulated in the 48 h old calves, the complements system, also upregulated at 48 h, and two interleukin signalling pathways, *1l-9* and *Il-12* in which the majority of genes were present at higher levels after 48 h. Investigating upstream analysis effector genes identified a number of significantly altered upstream regulators including a number of growth factors (*Areg, Hgf, VEGFA)*, cytokines *(c5f2, PRL, IFNa2)*, and also the Fc gamma receptor upstream pathway activated in the CL calves at 48 h. Supplementary Fig. 10 shows activation of transmigration of cells with many immunologically important receptors highlighted as upregulated in the 48 h calves. The calves were then compared at the later timepoint of 168 h to 72 h, to investigate the transition of the immune response evident through the blood transcriptome from day. Only 383 DEG were identified between the two time points in the CL calves. Top molecular cellular and physiological networks again included cellular movement, immune cell trafficking and also cellular function and maintenance. There were no canonical pathways identified as significantly enriched in the comparison. Overall a major stabilization of the immune response was evident through the inhibition of a number of immune related regulators (Supplementary Fig. 11).

The LF calves were also compared across the four time points (0 h, 48 h, 72 h and 168 h) to investigate the development of immunocompetence during the first week of life. Similarly high levels of DEG were evident at the early timepoint comparison of 48 h versus 0 h, with 2021 DEG identified in total, with 1640 of these being successfully mapped in IPA. Top enriched molecular and cellular networks included cellular movement, cellular function and maintenance; immune cell trafficking, and lymphoid tissue structure and development. A number of upstream regulators including a large number of cytokines (*TNFα, Il1β, IFN)*, transmembrane receptors (*TLR3, TLR4, TLR7, TLR9)* exhibited activation in the 48 h calves compared to 0 h.

**Figure 2:**
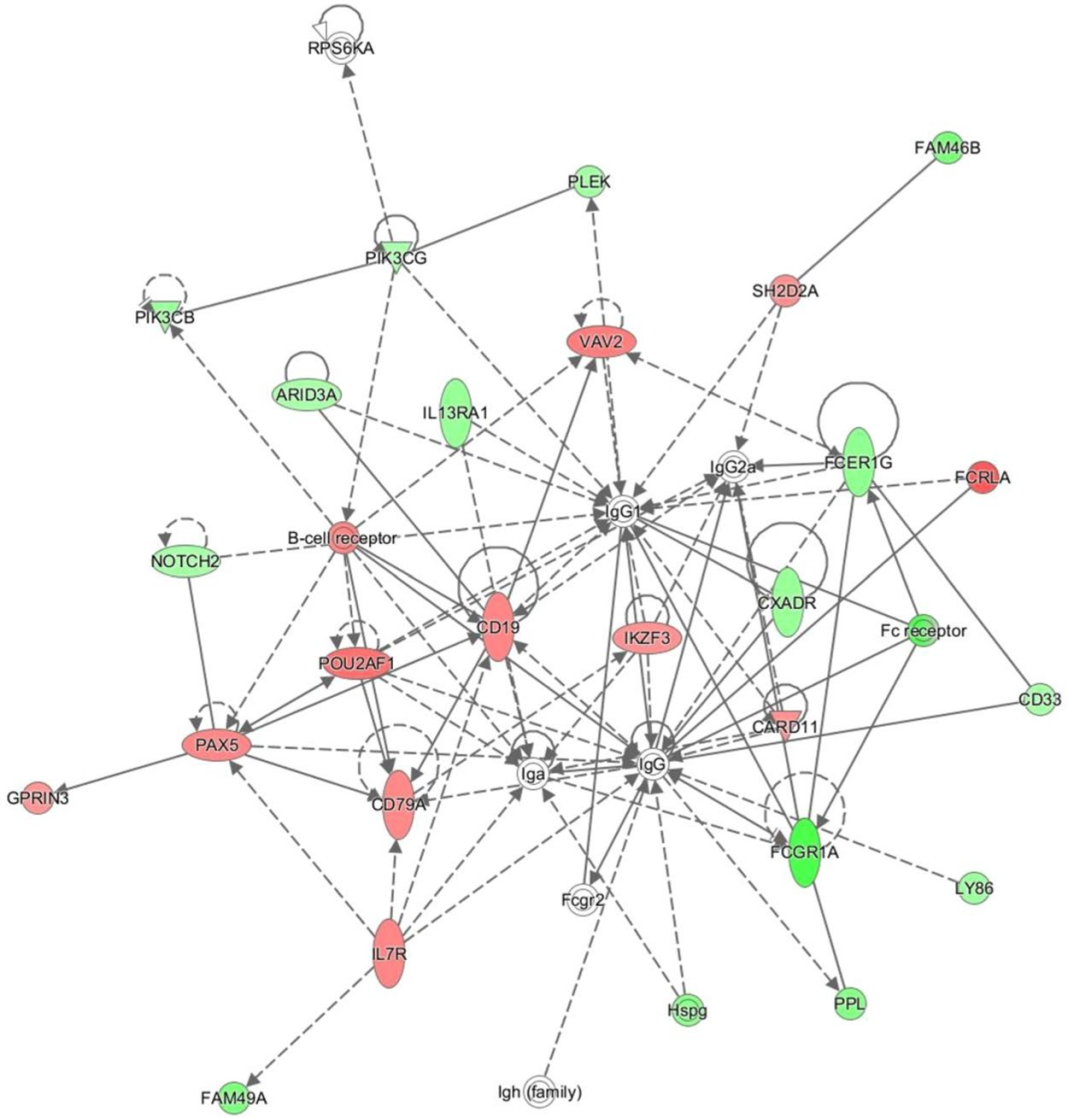
Humoral Immune response network enriched in comparison of Beef LF 168 h v Beef LF 72 h. Network 23- Humoral Immune Response, Protein Synthesis, Cellular Development network enriched in comparison of beef LF 168 h versus Beef LF 72 h. The network is displayed graphically as nodes (genes). The node colour intensity indicates the expression of genes; with red representing up-regulation and green, down-regulation in 168 h beef LF calves compared to 72 h beef LF calves.

**Figure 3:**
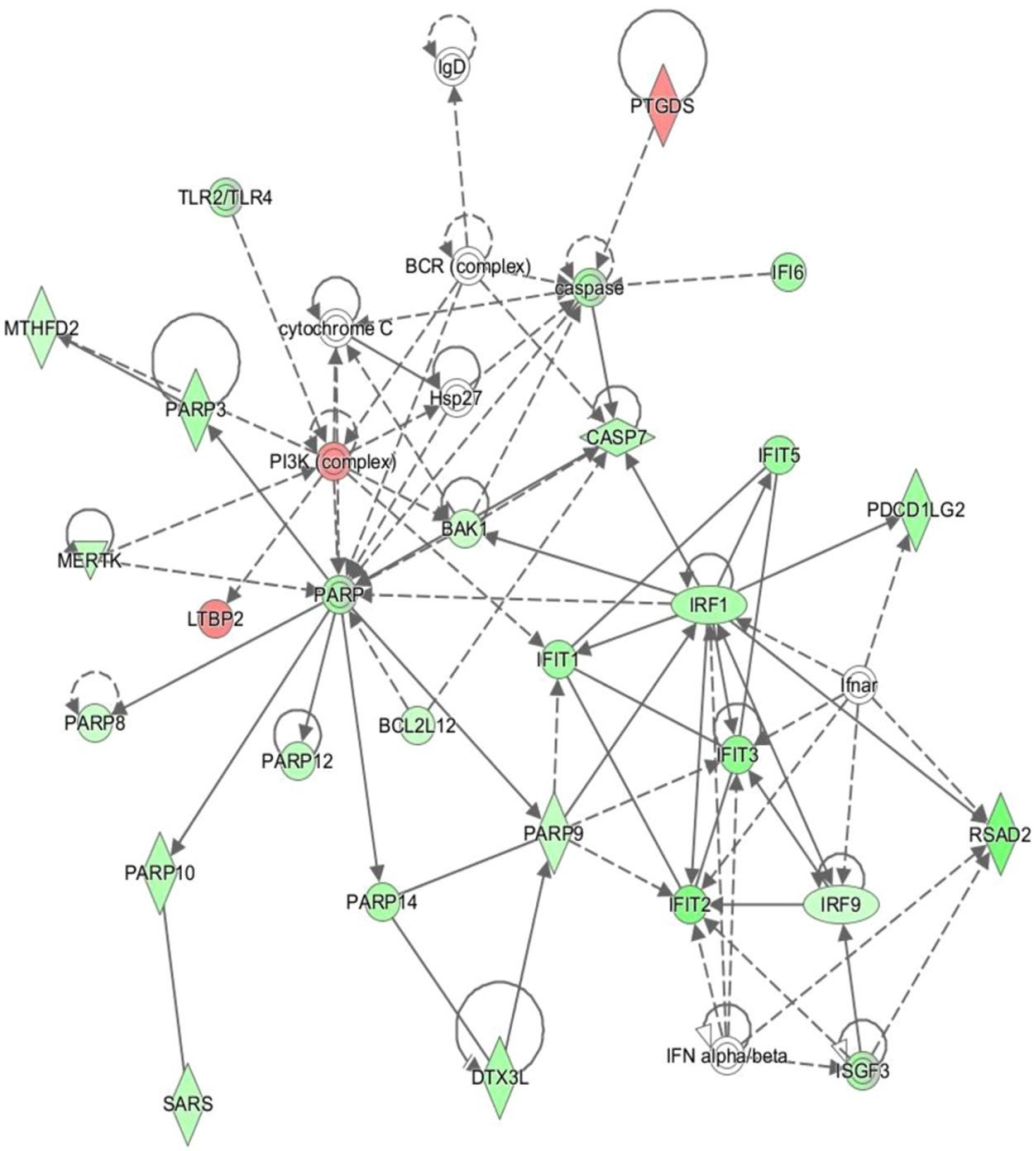
Infectious Diseases network enriched in comparison of Beef CL 48 h v Beef LF 48 h. Network 5- Infectious Diseases, Antimicrobial Response, Inflammatory Response network over enriched in comparison of beef CL 48 h and beef LF 48 h calves. The network is displayed graphically as nodes (genes). The node colour intensity indicates the expression of genes; with red representing up-regulation and green, down-regulation in 48 h beef CL calves compared to 48 h beef LF calves.

**Figure 4:**
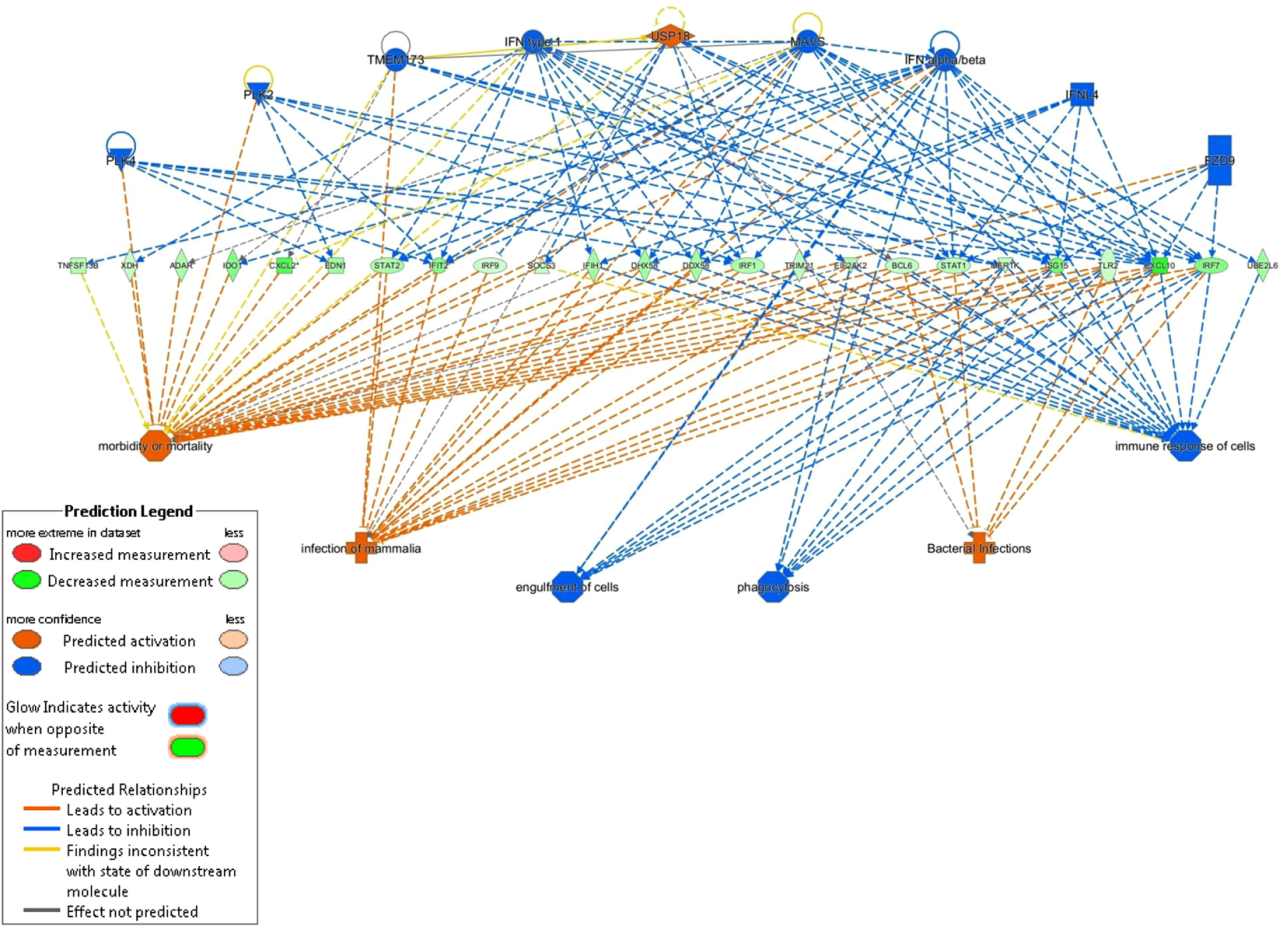
Regulator effects in comparison of Beef CL 48 h and Beef LF 48 h. Bacterial Infections, engulfment of cells, immune response of cells, infection of Mammalia, morbidity or mortality, phagocytosis regulator effects network. Image highlights predicted activation (orange lines) of bacterial interactions, morbidity/mortality, and infection of Mammalia in the Beef CL 48 h compared to Beef LF 48 h, with predicted inhibition of engulfment of cells and phagocytosis, and immune response predicted to be inhibited (blue lines) at the 48 timepoints in beef CL v beef LF.

LF calves were then compared at 168 h versus 72 h to identify observable alterations to key genes and pathways involved in the development of immunocompetence. Overall there were 1897 DEG identified, with 1562 being successfully mapped using IPA for functional analysis. The vast majority of total DEG, 1362 out of 1897, were present at lower levels in the 168 h calves, highlighting the stabilization on the initial surge in immune gene expression. A number of biologically interesting networks were enriched in pathway analysis of the 168 h and 72 h calves, including network 14 mapping enrichment of immune cell trafficking and the inflammatory response, of which the majority of genes are present at lower levels of expression by 168 h (Supplementary Fig. 12). Network 23 however, humoral immune response, showed upregulation of a number of genes in the 168 h calves (See Fig. 2). A significant number of upstream gene regulators including cytokines (*Il1α, IFNγ, Il1P, OSM)*, and immune regulatory complexes (*MAP2K1, NFκB)* were inhibited by 168 h.

(ii) Comparison of CL calves to LF calves across time: In order to investigate any differences in the development of a competent immune response during the first week of life in the two beef crossbreeds, the breeds were compared at each time point. Only the 48 h timepoint comparison generated a significant number of DEG, 716 in total, 594 of which were mapped for functional analysis using IPA. A number of interesting networks were highlighted as being enriched in the comparison of 48 h, including network 5, antimicrobial response, in which the majority of the genes were present at lower levels in the CL calves (Fig. 3). Figure 4 highlights variations in the overall immune response of cells showing inhibition in the CL calves at 48 h compared to the LF breed. Biologically important upstream regulators were also identified as being significantly differentially expressed in the comparison of beef CL to LF, including *NFκB* and *Il12* complexes, both inhibited in CL calves compared to LF at 48 h. A number of immunologically important upstream regulators were also inhibited in the beef CL calves, including *CD40LG*, the cytokine that binds to CD40 (Supplementary Fig. 13).

## Discussion

This study examined the immune-competence development of artificially fed dairy calves and naturally suckled beef calves from birth to 7 days of age using a whole blood transcriptomics approach. To date, no research has characterized, using a functional genomics approach, the immune transcriptome of breeds of calves, namely, dairy and beef, over the first 7 days of life under varying management systems. The contrast between dairy and beef breeds is also important as in some countries replacement breeding heifers for the beef cow herd are often sourced from the dairy herd i.e. beef × dairy cows. Below, we discuss alterations in the whole blood transcriptome over time from birth in a side by side comparison of dairy calves and in two beef crossbreeds. The whole blood transcriptome of the two beef crossbreed calves were also compared to each other across the three critical time points, to determine any differences in the transcriptomic profile that may indicate differences in development of immunocompetence in beef breeds.

Colostrum management on both beef suckler and dairy farms is of critical importance to the prevention of calf morbidity and mortality. It is generally perceived that the passive immune status of suckler-bred calves is superior to dairy-bred calves. This is evident under controlled research farm conditions, where it is ensured that calves suckle the dam and/or are fed sufficient colostrum in a timely manner post-parturition^5,7^. However, as the majority of dairy calves are removed from the dam at birth or shortly after, and artificially fed colostrum, management practices are of particular importance. First milking colostrum, fed within two hours of birth is widely recommended, but of equal importance is the volume of colostrum fed. Research suggests that 10% of total body weight should provide sufficient volumes of colostrum for dairy calves, but management practices under artificial feeding systems means that this may not always be the case^14–16^. For the purpose of this study, 5% total body weight of colostrum was artificially fed to dairy calves, with both beef breeds ingesting colostrum from the dam under observation, in order to identify alterations to critical genes during the first week of life.

Firstly, the transcriptomic profile of dairy calves over the first week of life was examined. Networks identified as over-represented in the comparison of 48 h and 0 h dairy calves included haematological system development and immune cell trafficking, although the genes involved in these networks did not display a consistent pattern of expression. Comparison of 48 h to 0 h dairy calves resulted in top canonical pathways including the role of pattern recognition receptors, G protein coupled receptor signalling and Il-17a signalling. The expression of the genes involved in pattern recognition at 48 h in the dairy calves however, was lower than at 0 h. The role of PRRs in the innate immune response is well documented and the decreased expression of genes involved in such a pathway in the dairy calves could potentially result in immune impairment^17,18^. Complement system however, was upregulated at 48 h in the dairy calves. *CF1*, which encodes complement factor 1 was present at increased levels of almost 3 fold higher at 48 h post birth. The complement system is a vital component of the innate immune response compromised of a large group of proteins that work together to opsonize microbes for clearance^19^. A significant number of cytokines and chemokines were present in altered levels of abundance in the dairy calves at 48 h post birth compared to o h. *CXCL10* was upregulated at 6.78 RFC in dairy calves at 48 hr, which is thought to play a role in T cell chemotaxis. T cells are regarded as a crucial link between the innate and adaptive immune responses^20,21^. Analysis of upstream regulating genes in the 48 h dairy calves compared to the 0 h calves identified a number of cytokines as being inhibited in this period at the later time point including *Il4* and *Il1β*, two immunologically important regulatory genes^22^. Chemokine expression was also reduced overall in the 48 h dairy caves when compared to the 0 h calves. Chemokines are involved in immune cell activation and chemotaxis and a reduced level of transcriptional abundance by 48 h could lead to immune impairment^23^. A significant number of solute carrier genes were down regulated at 48 h in the dairy calves compared to 0 h. Solute carrier proteins are responsible for the transport of sodium and bicarbonate ions across the cell membrane. The significant reduction in such a large number of these genes is not completely clear but could be due to the fact that colostrum has a much higher sodium content than transition milk and mature milk^24^. The high sodium content may result in a necessary upregulation in ion transporters, which are then present at lower levels of expression by 48 h.

At 168 h post birth, the transcriptome of the dairy calves was compared to that at 72 h, to elucidate expressional changes of critical genes and monitor possible progression towards immunocompetence. In dairy calves, over-represented networks at the 168 h *vs* 72 h time points are mostly those involved in cell cycle, maintenance and growth. There was no clear indication of a major immunological change in the dairy calves through observation of the transcriptomic profile at 168 h. Chemokine expression however, was upregulated in the 168 h compared to the earlier time points. Top upstream regulators included *CSF2* which encoded granulocyte stimulating factor; a cytokine that stimulates the differentiation of hematopoietic precursor cells such as granulocytes and macrophages^25^. Overall, there is an observed downregulation of members of the interleukin superfamily at 168 h post birth in the dairy calves expression however, was down regulated at 168 h in the dairy calves, including *IL11RA* and *IL1F10*. The downregulation of interleukins at 168 h could potentially have detrimental effects on the immune response, as they are particularly important in stimulating immune responses, such as inflammation.

Following transcriptional analysis of the development of a competent immune response in dairy calves over the first week of life, beef calves were then examined in a similar manner. In beef LF calves, comparison at 48 h versus 0 h highlighted the role of pattern recognition receptors (PRRs) as being over-represented, with the majority of genes upregulated at 48 h. The most noticeable increase to the expression of cytokines was observed in the LF crossbreed calves, with a large number of cytokines present at increased levels of abundance by 48 h. *CXCL10* was present at 4 RFC and 28 RFC in the beef CL and LF calves respectively, at 48 h compared to 0 h. *CXCL10* encodes a small IFNγ inducible pro-inflammatory cytokine linked to having significant function within the immune response, involved in T cell chemotaxis and in monocyte migration among many proposed functions^26^. *CF1*, an important component of the complement defence system was also present at higher levels in both the LF and CL beef crossbreeds, at 5 fold and 7.2 fold higher respectively at the 48 h time point, similar to the increase seen in the dairy calves over the first 48 h post birth. Significant alterations to the molecular transport system are evident across both beef breeds at 48 h following birth and colostrum ingestion. Interestingly, the three most down regulated genes across beef CL and beef LF were the same; *SLC26A8, IL1R2*, and *PTX3. SLC26A8* is a solute carrier protein as previously discussed, and is also the most down regulated gene in the dairy calves at 48 h. *IL12*, is a decoy non-signalling receptor found on B cells, neutrophils, monocytes and macrophages, and is responsible for immune regulation through the capturing of Il-1^27,28^.

In beef calves, the top enriched pathway identified in the comparison of 168 h and 72 h calves included the complement system, over-represented in CL. The majority of additional over-represented networks and pathways within the beef CL calves had the majority of genes down regulated at 168 h such as *Il8* and *Il12* signalling. LF calves on the other hand appear to have an abundance of networks and pathways involved in the developing immune response at the one week stage, including pathways involved in Th1 and Th2 activation, and also in T helper cell differentiation. In addition to the over-representation of these immune pathways, LF calves also highlight an important gene, *JCHAIN*, as the second most abundantly expressed gene at the 168 h time point. This gene encodes joining chain of multimeric IgA and IgM, and could be indicative of the early signs of a developing adaptive immune response, not present at a detectable level of transcription as of yet in CL calves^29,30^.

In order to fully elucidate immune differences between the two beef crossbreeds, CL transcriptomic profiles were compared to LF at each time point, which was possible due to similar management in both breeds. Only the 48 h time point gave significant levels of DEG for functional analysis, which in itself is of interest, as this time point is the most critical in the observation of possible immune alterations, following colostrum consumption. There is evidence to suggest that LF dams produce higher quality colostrum^3^. Overall, the majority of immunologically important pathways contained genes that were present at lower levels in the CL calves, including interferon signalling, an infectious disease network, and an overall antimicrobial response network. A number of upstream regulators were also present at decreased levels of expression in the CL calves at 48 h compared with LF calves, including *LPS*, *IRF7*, and *IFNα*. The decreased levels of expression across these key immune gene regulators is suggestive of a lower immune capability in CL calves at 48 h compared with LF. The expression of a group of chemokines were also downregulated in the CL calves at 48 h compared to LF. Almost all of the genes present at the lowest levels of abundance in the CL calves when compared to LF at 48 h were immune related genes, such as *CD274*, which encodes an immune inhibitory receptor ligand that is expressed by hematopoietic and non-hematopoietic cells, such as T cells and B cells. The gene present at the lowest level of mRNA abundance in CL calves at 48 h was *MAL*, which encoded a T cell differentiation protein^31^. These data provide some further evidence that the colostrum quality of the LF dams may have higher benefits over that of CL.

### Concluding remarks

Here we observed for the first time, the development of an immune response in calves of different breeds over the first seven days of life, using whole blood and RNA sequencing technologies. The molecular variations in key genes such as cytokines and B cell receptors across all breeds gives a tremendous insight into possible reasons as to why certain cattle breeds are more susceptible than others to illness during the neonatal period. It is clear from these data, that dairy calves undergo a systemic stress response following separation from the dam at birth and following artificial feeding of colostrum. Key genes increased in LF calves at 168 h including the immunoglobulin receptors *FCER1A* and *FCRLA*, and *IL-12r*, an immune regulator consistently down regulated in beef CL and in dairy calves, offer possible targets for biomarker discovery and a greater understanding into the development of immunocompetence.

## Materials and Methods

All animal procedures performed in this study were conducted under experimental licence from the health products regulation authority (HPRA), Dublin, Ireland (individual authorisation number, AE 19132/I073; project licence number, AE 19132/P006) and the United Kingdom (UK) Animals (Scientific Procedures) Act 1986. Dairy calves were sourced from the Agri-Food and Bioscience Institute (AFBI) research dairy farm in Hillsborough, located in Co. Down, Northern Ireland (latitude 52°27′, longitude 6°4′) and beef calves were sourced from the beef suckler herd, at Teagasc Grange, located in Co. Meath, Ireland (latitude 53.52187, longitude −6.65247).

### Animal model

The animal model was designed to fully elucidate the development of immunocompetence in i) Holstein dairy calves (artificially fed colostrum at a level of 5% total body weight (BW) at birth) and ii), beef suckler calves, which were the progenies of Limousin × Friesian and Charolais × Limousin cows. The cows were bred using Aberdeen Angus (AA) and Charolais (CH) sires (natural mating) (LF calf progenies 3 AA and 4 CH; CL calf progenies 3 AA and 4 CH).

The birth of each dairy calf was vigilantly observed and calves were separated from their dam approximately 15 to 20 min post-birth to prevent suckling occurring. Calves were weighed and placed in a straw-bedded pen where they were blood sampled and received their first feed of colostrum, fresh from their own dam, (at a concentration of 5% total body weight (BW)) via oesophageal tube within 2.5 h post birth. 12 h later, calves were fed 5% body weight, second milk colostrum, via a teat feeder. Calves were fed 12.5% of their BW of colostrum from their own dam for the first 4 days of life. Calves were weighed at birth and 70 d using a calibrated weigh scale (Tru-Test Eziweigh 5, Auckland, New Zealand). Calves were individually penned for the first 4 days of life, and on day 5 were placed in a group pen where they were fed a skim based calf milk replacer (protein and oil content of 23% and 19% respectively) (Britmilk, Dumfries, UK) (Supplementary Table 1) and offered *ad libitum* concentrate and clean water from an automatic milk feeder (Forster Technik vario, Germany). Once calves were introduced to the automatic feeder on d 5, their weights were recorded on a daily basis using an automated weigh scale until d 63 (Forster Technique, body scale, Germany). Two beef cow breeds were included: Limousin × Friesian (LF) (n = 7) and Charolais × Limousin (CL) (n = 7). Mean parity (SD) for LF and CL beef suckler cows was 5.5 (0.94) and 5.3 (1.03), respectively. The mean calving date was 28^th^ February 2015 (26^th^ January to 27^th^ April). All calves involved in the study were single birth calves and had a mean calving difficulty score of 2.1, ranging from 1 (unobserved/unassisted) to 3 (assisted with calving aid). Births were supervised by farm personnel to ensure that calves did not suckle the cow prior to implementing the experimental protocol for collection of the first blood sample from the calf. All calves were closely observed to ensure suckling of the dam and any calf not suckling within 1 h of birth or showing signs of weakness was assisted to suckle the cow. After parturition, beef calves remained with their dams and had free access to their dams for suckling until weaning after 7 months of age. Calf live weights were recorded at birth, and every four weeks thereafter throughout the experimental period. Mean (SD) calf birth weight was similar (P > 0.05) for the progeny of CL (46.4 (4.74) kg) and LF (46.6 (6.27) kg) cows. The live weight gain of calf progenies from LF cows was greater from birth to weaning (P < 0.05) compared to the calves from CL cows.

#### Cow vaccinations

All beef and dairy cows were immunised against the inactivated antigen strain of BoHV-1 (gE-) with a primary vaccine and a secondary booster vaccine at day (d)-84 and d-56 relative to the expected calving date (d 0), with 2 ml (S/C) of a commercial vaccine, Bovilis IBR marker inactivated (MSD Animal Health, Buckinghamshire, UK). All cows received a combined rotavirus, coronavirus and *E. coli* F5 (K99) vaccine (inactivated) (Rotavec Corona) by intra-muscular injection between 4 and 12 weeks prior to calving date.

#### Colostrum milk collection and sampling

One 50 mL sample of colostrum was taken at calving, prior to the initial feed. Samples were collected from the left quarter of the dams’ udders. All quarters of the udder were cleaned before sample collection with dry paper towel, to avoid any debris entering the sample. Colostrum samples were analysed for total IgG. The 50 mL colostrum samples were centrifuged at 2000 × *g* at 7 °C for 10 minutes. Following centrifugation, the fat-layer was removed with a sterile spatula. The supernatant (fat-free) was frozen at −20 °C for IgG analysis^32–34^.

#### Blood collection

Blood samples were collected from the calf *via* the jugular vein using an 8.5 ml BD vacutainer (SST tube) (BD, Oxford, UK), at 0, 48, 72, and 168 hour (h) post birth. All blood samples were stored at room temperature for a minimum of 1 h to allow clotting to occur. Samples were then centrifuged at 1764 *g* for 15 min, the serum was collected and subsequently stored at −20 °C for IgG analysis.

Bloods for RNA extraction were collected via jugular venipuncture from all calves at 0 h, 48 h, 72 h, and 168 h post birth. The blood (3 mL) was collected using tempus blood RNA tubes (Life technologies, Paisley, Scotland). Immediately after blood collection, the tubes were shaken vigorously for 10 s, followed by storage at −80 °C until RNA isolation.

### Colostrum and serum IgG analysis

Colostrum (fat free) and serum (calf) samples were thawed at 4 °C overnight. IgG concentration was then determined using an enzyme-linked immunosorbent assay (ELISA) kit for bovine IgG from Bio-X Diagnostics (Jemelle, Belgium)^32,33^. All components of the kit were brought to room temperature (21 °C) before use. The wash buffer was diluted 20 fold with distilled water. A calibration curve was constructed and all samples were diluted at 1:225 in phosphate buffer saline (PBS), as per manufacturer’s instructions. All samples (100 µl) were added to the test plate and tested in duplicate, along with standards using a 96-well dilution plate. Equal volumes of horse radish peroxidase (HRP) conjugate was added to each well of the dilution plate and then 100 µL of this was transferred to the test plate. Each test plate was incubated at room temperature (21 °C) for 1 hour. The plate was washed three times using wash solution. Chromogen solution (tetramethylbenzidine) was added to each well (100 µl/well) and the plate was protected from light and incubated for 10 minutes at room temperature. Stop solution was added to each well (50 µl/well) and the absorbance (optical densities) of the samples was read at a wavelength of 450 nm using a microplate spectrophotometer (Tecan Magellan, Männedorf, Switzerland). Negative controls were included on the test plate. An inter-assay CV of <0.15 was observed. These concentrations of IgG in the samples were averaged and then subtracted from the known concentrations of IgG in the reference standard absorbance values provided in the test kit. A standard curve was produced using the average values from duplicate standard wells. This curve illustrated the relationship between absorbance and IgG concentration. The coefficient of determination value for each assay had to exceed 0.9 on the standard curve for inclusion within these results^32–34^.

### RNA Isolation and Purification

RNA was isolated from whole blood collected from dairy and beef calves using the tempus spin RNA isolation reagent kit following manufacturer’s guidelines (Applied Biosystems, USA). The quantity of the RNA isolated was determined by measuring the absorbance at 260 nm using a Nanodrop spectrophotometer ND-1000 (Nanodrop Technologies, DE, USA). RNA quality was assessed on the Agilent Bioanalyser 2100 using the RNA 6000 Nano Lab Chip kit (Agilent Technologies Ireland Ltd., Dublin, Ireland). RNA samples with 28S/18S ratios ranging from 1.8 to 2.0 and RNA integrity number (RIN) values of between 8 and 10 were deemed to be of high quality. RNA was stored at −80 °C until subsequent cDNA library preparation.

### cDNA library preparation and sequencing

cDNA libraries were prepared using the Illumina TruSeq RNA sample prep kit following the manufacturer’s instructions (Illumina, San Diego, CA, USA). For each sample, 0.7 μg of RNA was used for cDNA library preparation. Briefly, mRNA was purified from total RNA and then fragmented. First strand cDNA synthesis was performed using SuperScript II Reverse Transcriptase (Applied Biosystems Ltd., Life Technologies) subsequently synthesising the second strand using components of the Illumina TruSeq RNA sample prep kit. Adaptors were ligated to the cDNA which was then enriched by PCR. Final individual cDNA libraries were validated on the Agilent Bioanalyser 2100 using the DNA 1000 Nano Lab Chip kit (Agilent Technologies Ireland Ltd., Dublin, Ireland), ensuring that library fragment size was ~100 base pairs (bp) and library concentration was >30 ng/μl. Subsequently, individual RNAseq libraries were pooled based on their respective sample-specific-6 bp adaptors and sequenced at 100 bp/sequence single-end read using an Illumina HiSeq2000 sequencer (Macrogen Europe, The Netherlands). Approximately 38.4 million sequences per sample (Mean ± SD = 38,432,764 ± 7,169,473) were generated, with each calf representing an individual sample.

### RNAseq data analysis

Reads were quality-filtered and adapter-trimmed using cutadapt, trimming the first 13 bp standard Illumina adapter, retaining reads with minimum base quality 30, minimum read length 20. Quality assessment of the filtered data was performed by FastQC. Both cutadapt and FastQC were called using the wrapper script TrimGalore! Using this information, samples with fewer than 10^7^ reads were rejected from further analysis. Reads were mapped against the UMD3.1 Bos taurus genome from Ensembl 87. Read alignment was performed using STAR. Gene level read-counts were quantified using STAR’s ‘–quantMode GeneCounts’ parameter. Analysis of the count data was performed using R’s Bioconductor package DESeq2. Raw count data was supplied to DESeq2, with no prior normalisation/transformation steps applied, as per DESeq2 guidelines. A DESeq2 internal analysis pipeline (DESeq) was then applied to the raw data, which estimates size factors, dispersion, performs negative binomial GLM fitting and calculates Wald statistics. Separately, read counts were transformed using DESeq2’s varianceStabilizingTransformation function for visualisation purposes. Hierarchical Clustering (HC) and Principal Components Analysis (PCA) were used in initial, unsupervised analysis of the transformed data to provide information regarding trends and potential outliers. HC analysis was applied using average linkage and Pearson correlation as a distance metric. DESeq2 was used to perform differential expression analysis on the output of the DESeq pipeline. The Benjamini and Hochberg adjusted p-value threshold to denote statistical significance of changes in a given gene’s expression levels was set to 0.05, with a fold change ±1.5 considered as a significantly altered level of transcript abundance. Genes above these requirement thresholds were referred to as differentially expressed genes (DEG) throughout.

### Pathway analysis

To examine the molecular functions and genetic networks, the RNAseq data were further analysed using Ingenuity Pathway Analysis (v. 8.8, Ingenuity Systems, Mountain View, CA; http://www.ingenuity.com), a web-based software application that enables identification of over-represented biological mechanisms, pathways and functions most relevant to experimental datasets or genes of interest^35,36^. Network eligible genes derived from these datasets were overlaid onto a global molecular network developed from information contained in the Ingenuity Knowledge Base. Networks of these genes were then generated algorithmically based on their connectivity (Ingenuity Systems, 2005). The network score is based on the hypergeometric distribution and is calculated with the right-tailed Fisher’s Exact Test. Canonical pathway analysis identified the pathways from the IPA library of canonical pathways that were most significant to the dataset in terms of the ratio of the number of genes that mapped to the pathway from the dataset and a right-tailed Fisher’s exact t-test to determine the probability that the genes mapped to the pathway by chance alone.

### Statistical analysis of serum IgG concentration

Colostrum and serum IgG data were checked for normality and homogeneity of variance by histograms, QQ-plots, and formal statistical tests as part of the UNIVARIATE procedure of SAS (version 9.4; SAS Institute, 2006). Data that were not normally distributed were transformed by raising the variable to the power of lambda. The transformed data were used to calculate P-values. The corresponding least squares means (Lsmeans) and SEM of the non-transformed data are presented in the results for clarity. The serum IgG data were analysed using repeated measures mixed model ANOVA (PROC MIXED, SAS v 9.4) with animal ID, breed (cow breed), sampling time and their interactions included as a fixed effect. REML linear mixed models, was performed to detect significant differences between experimental breeds. The statistical model consisted of the individual animal as the experimental unit in all analyses. Breed was the fixed effect within the model. Animal was included in each model as a random effect. For repeated measures, sampling time was included as the repeated measure. Non-statistically significant interactions were removed from all the models. The type of variance-covariance structure used was chosen depending on the magnitude of the Akaike information criterion (AIC) for models run under compound symmetry, unstructured, autoregressive, heterogeneous 1st order autoregressive, or Toeplitz variance-covariance structures. The model with the least AIC value was selected. The differences between mean values were determined by F-tests using Type III sums of squares. The PDIFF was applied as appropriate to evaluate pairwise comparisons between means. A probability of P < 0.05 was selected as the level of significance and statistically tendencies were reported when P < 0.10. Differences between the means were examined using the PDIFF (difftype) option within the MIXED procedure of SAS. Means were considered statistically significant at a probability level of P < 0.05.

## Electronic supplementary material

Supplementary Figures 1-13

Dataset 2

Dataset 3

Dataset 4

Dataset 5

Dataset 6

Dataset 7

Dataset 8
