# Supplementary material for: Blood immune transcriptome analysis of artificially fed dairy calves and naturally suckled beef calves from birth to 7 days of age
Source: Sci Rep. 2018 Oct 18;8:15461. doi: 10.1038/s41598-018-33627-0 (PMC6194081; doi:10.1038/s41598-018-33627-0)
Supplement: Supplementary file 1 — Supplementary Figures 1-13 [file 41598_2018_33627_MOESM1_ESM.pdf]

Supplementary Figures 1-13

Blood immune transcriptome analysis of artificially fed dairy calves and naturally suckled beef calves from birth to 7 days of age

C. Surlis[1*], B. Earley[1], M. McGee[1], K. Keogh[1], P. Cormican[1], G. Blackshields[1], K. Tiernan[1], A. Dunn[2], S. Morrison[2], A.Arguello[1], S. M. Waters[1]

Supplementary Figure 1

Comparing Dairy 5% over time

D.05.048 V D.05.000

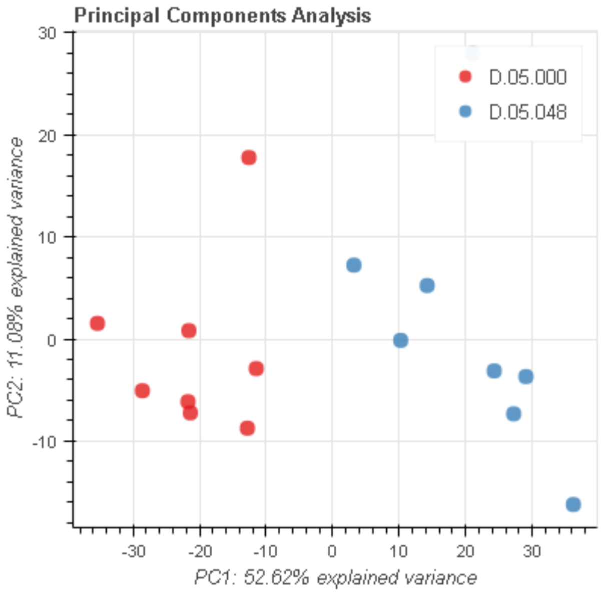

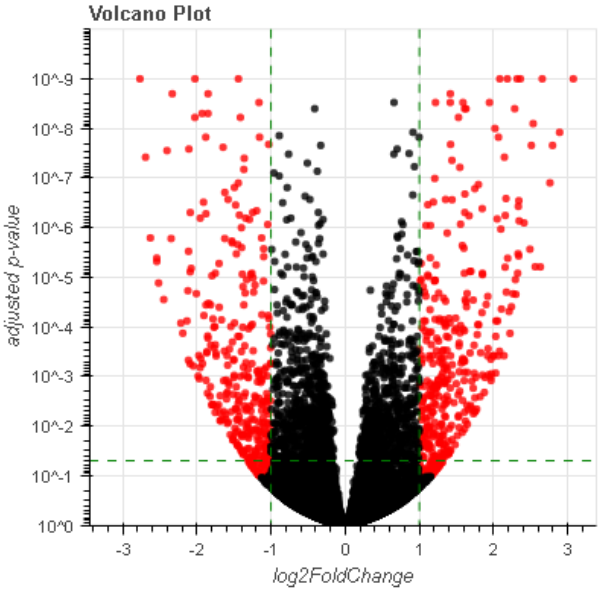

D.05.168 V D.05.072

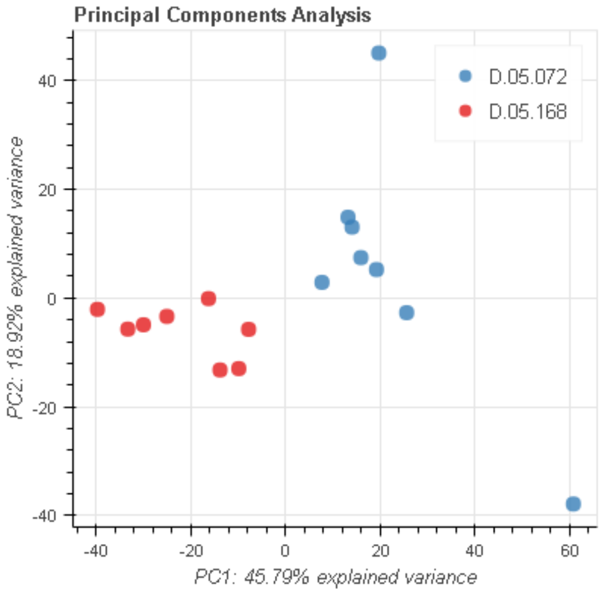

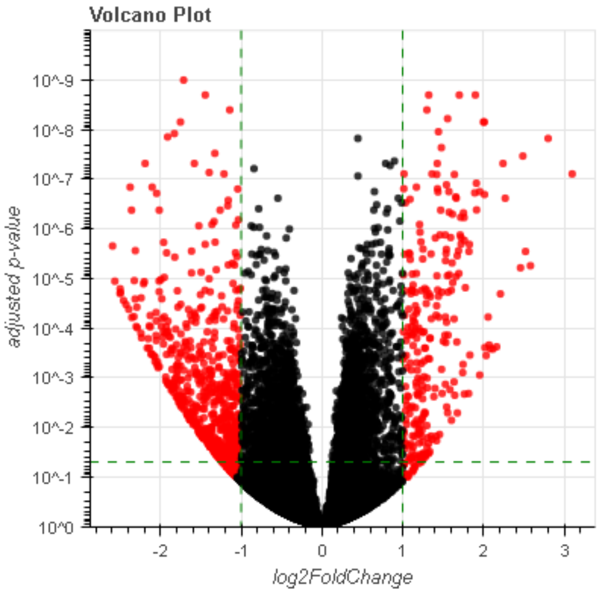

Supplementary Figure 2

Comparing Beef Breeds

Beef LF V Beef CL across timepoints

B.CL.048 V B.LF.048

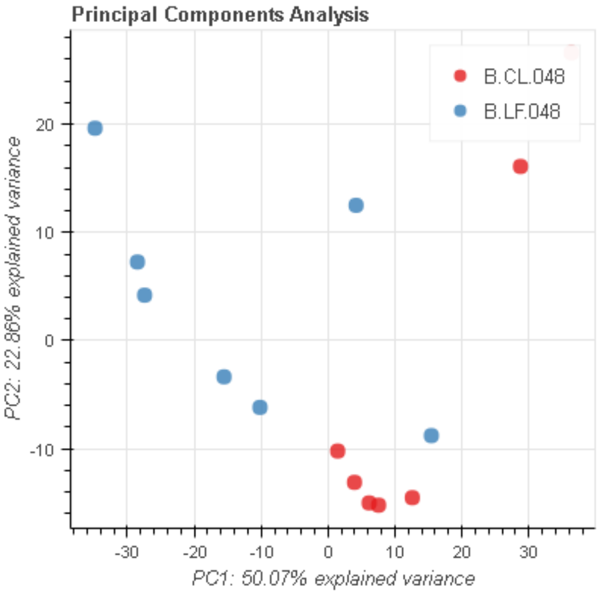

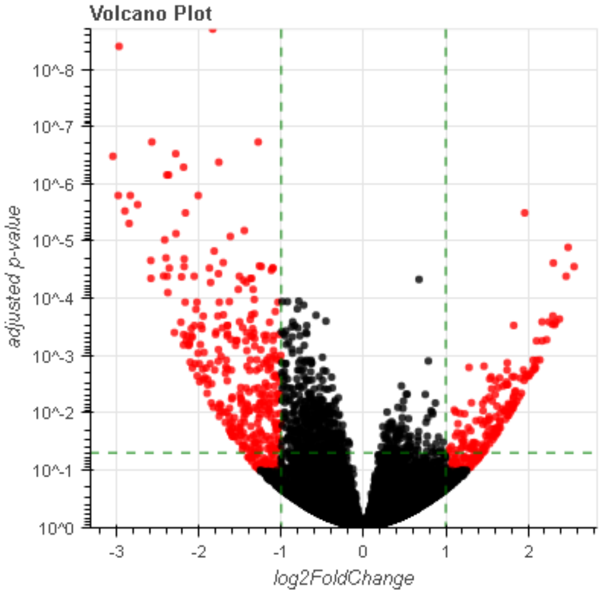

Supplementary Figure 3

Beef LF across time

B.LF.048 V B.LF.000

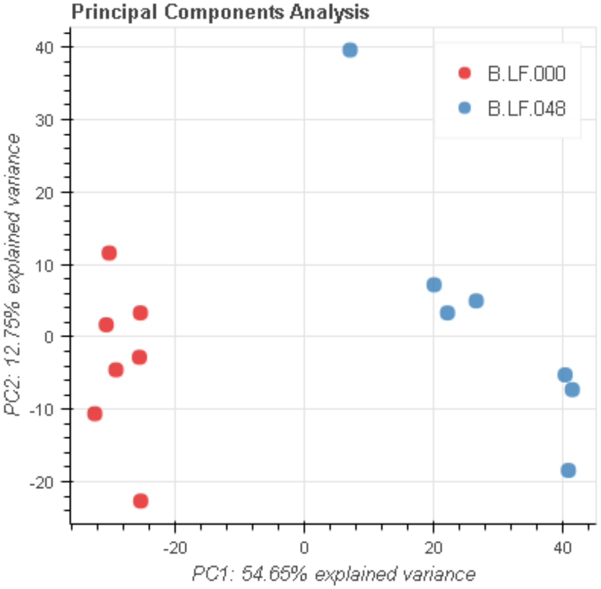

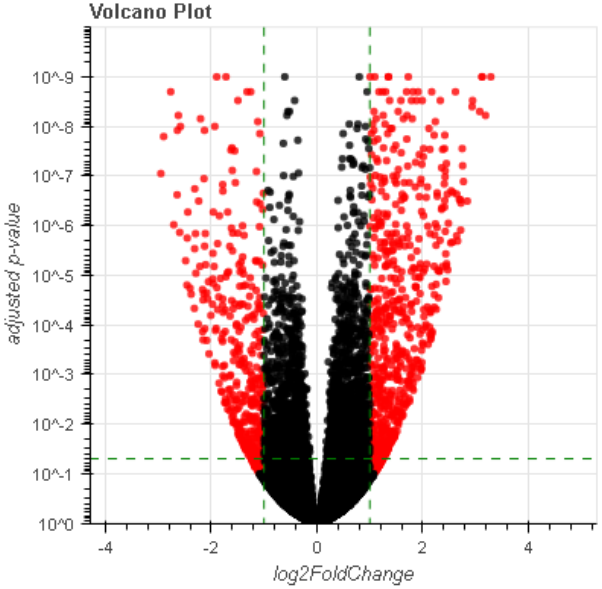

B.LF.168 V B.LF.072

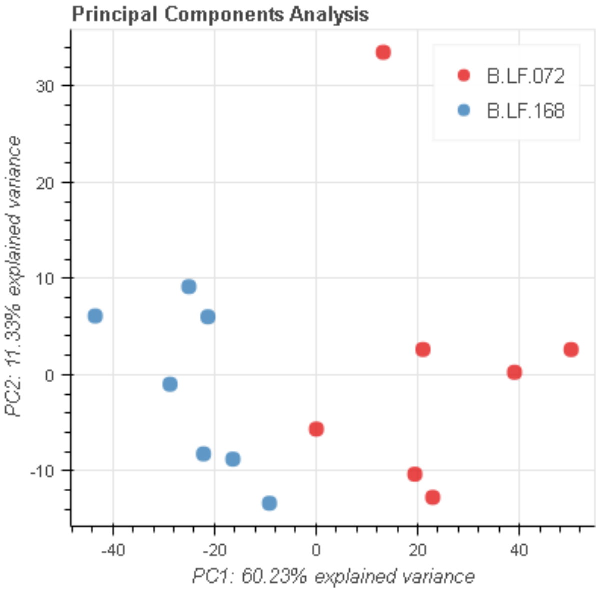

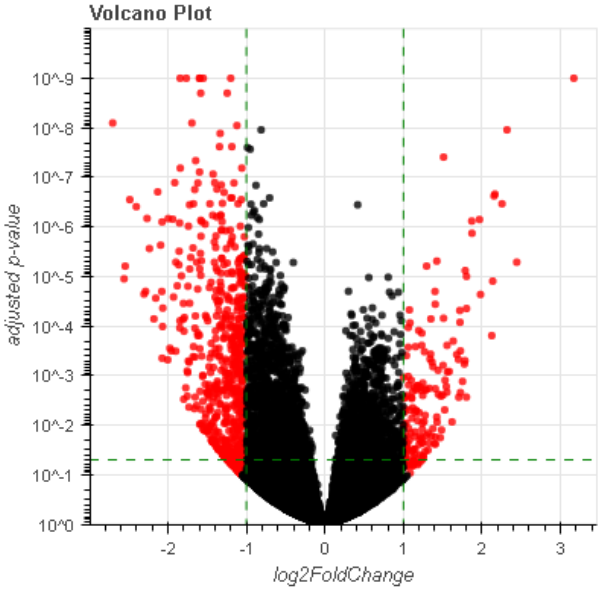

Supplementary Figure 4

Beef CL across time

B.CL.048 V B.CL.000

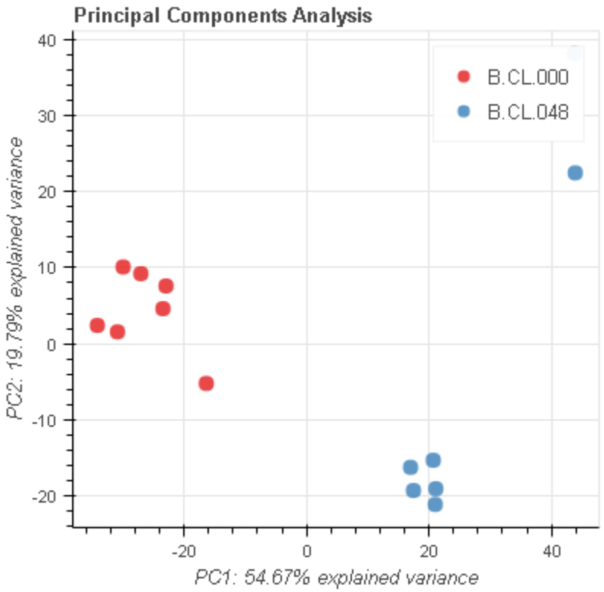

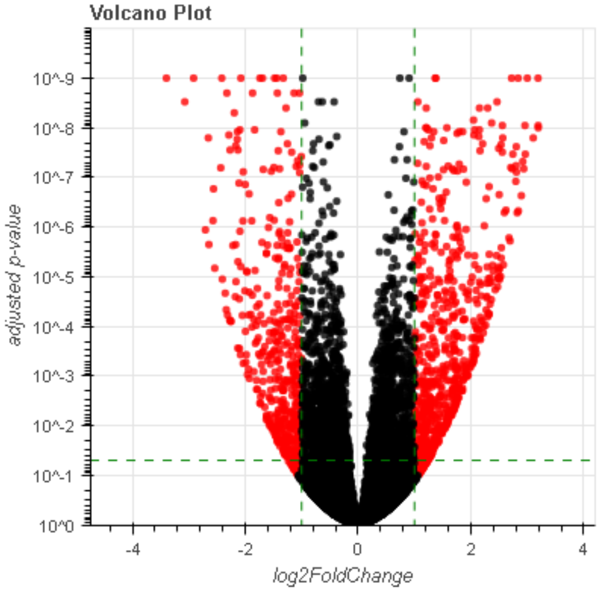

B.CL.168 V B.CL.072

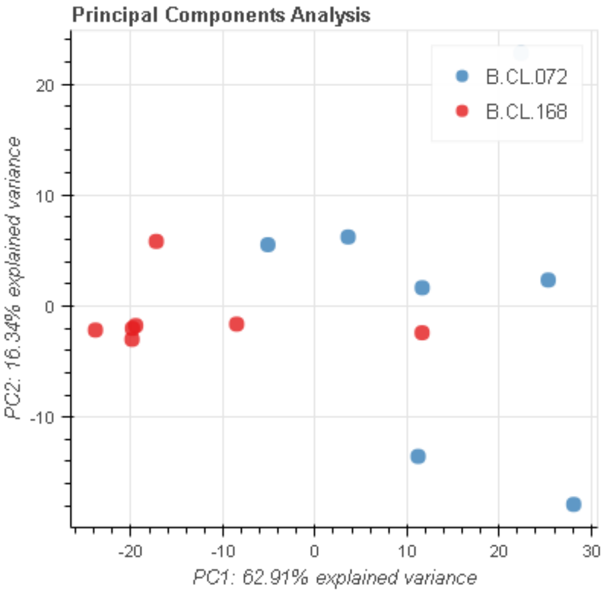

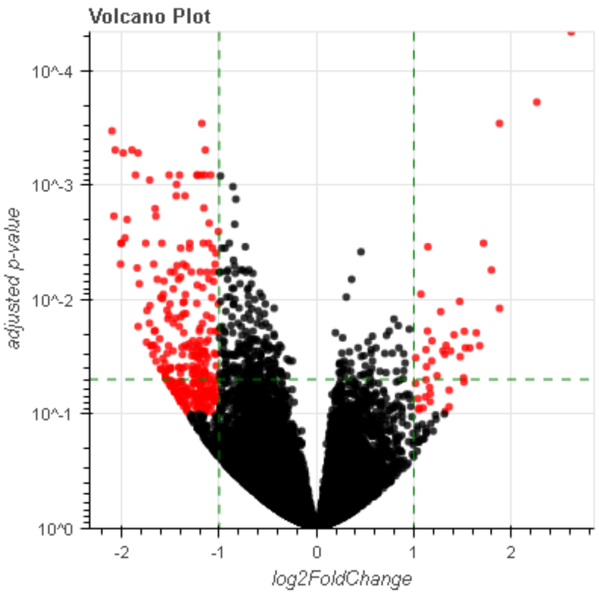

Supplementary Figure 5

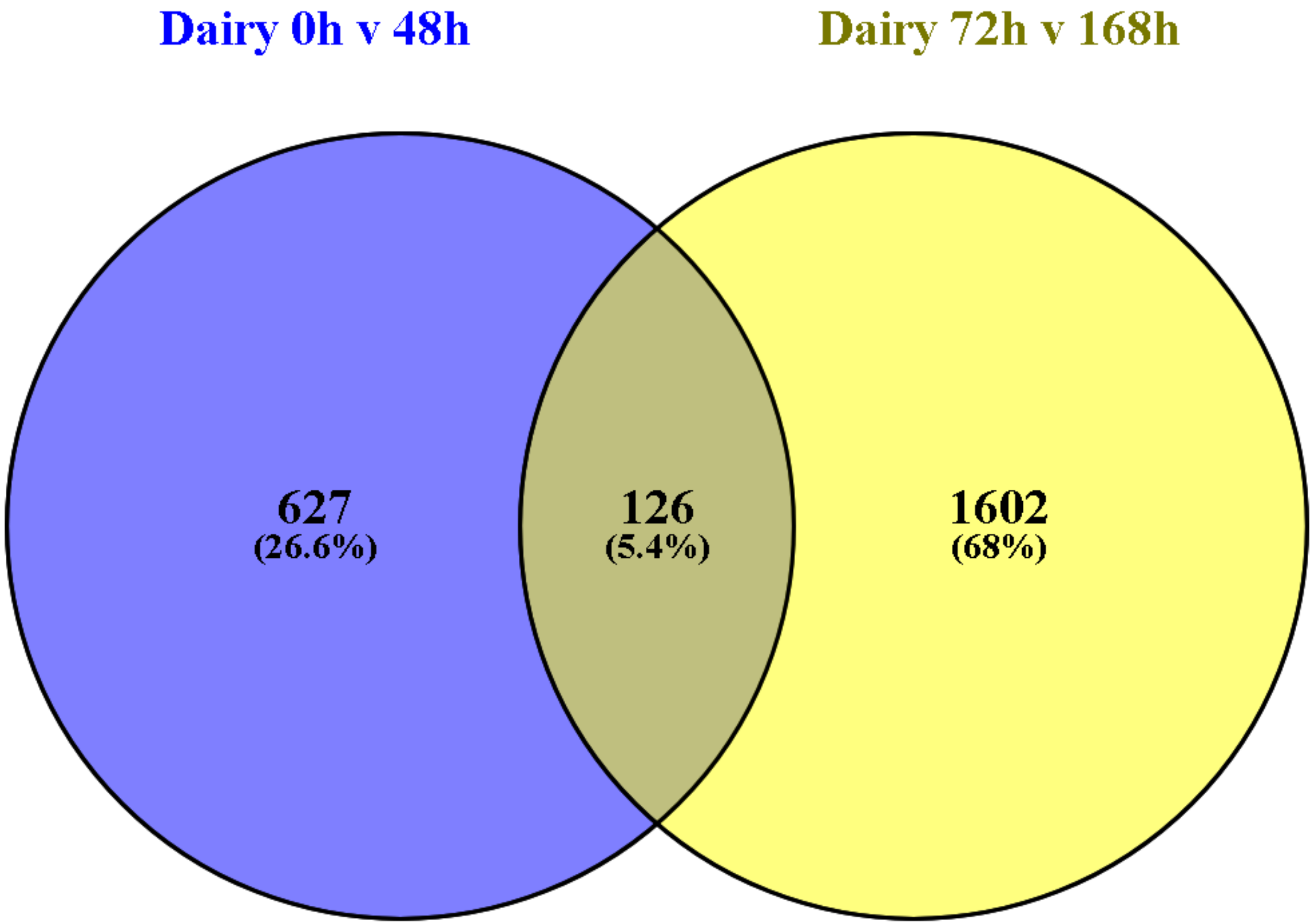

Supplementary Figure 6

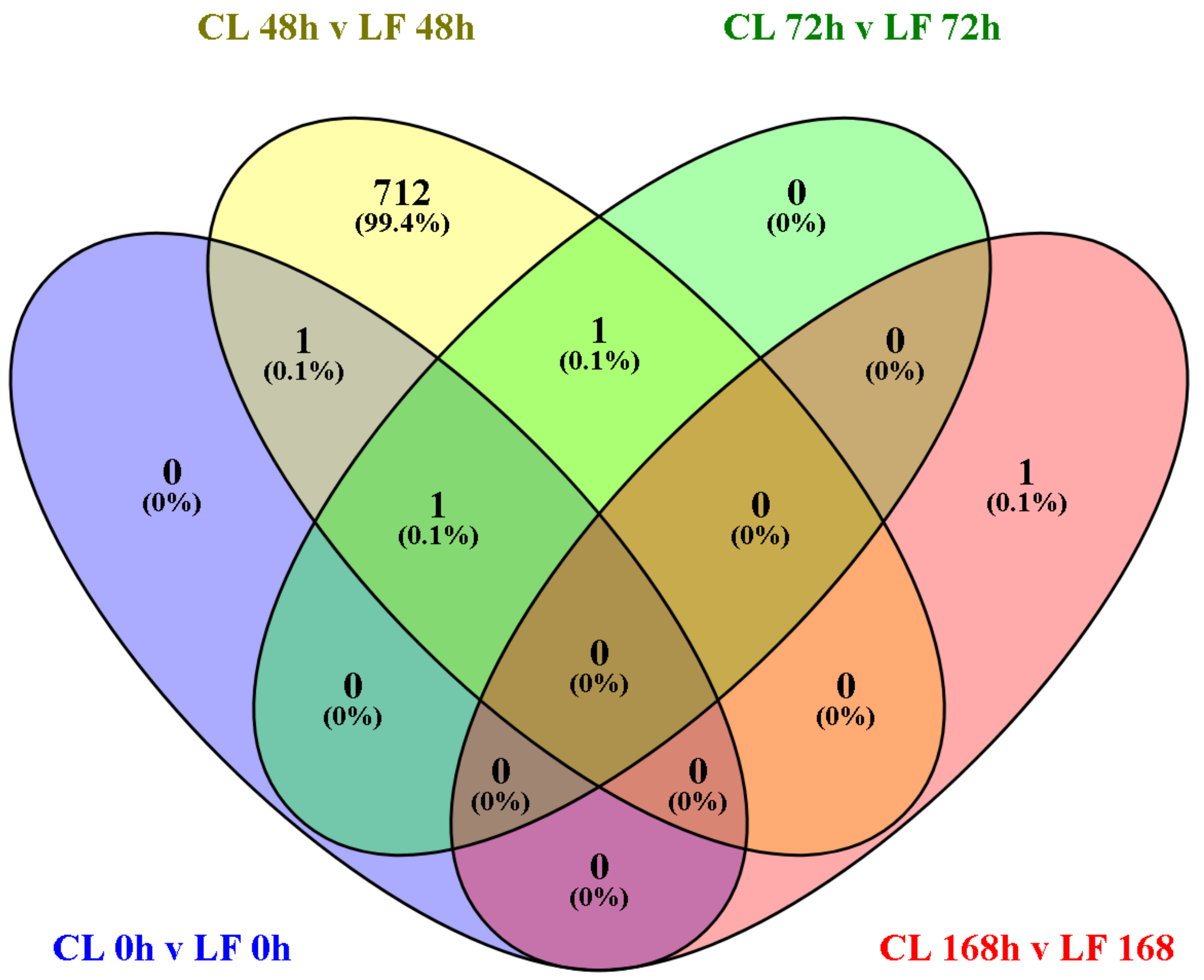

Supplementary Figure 7

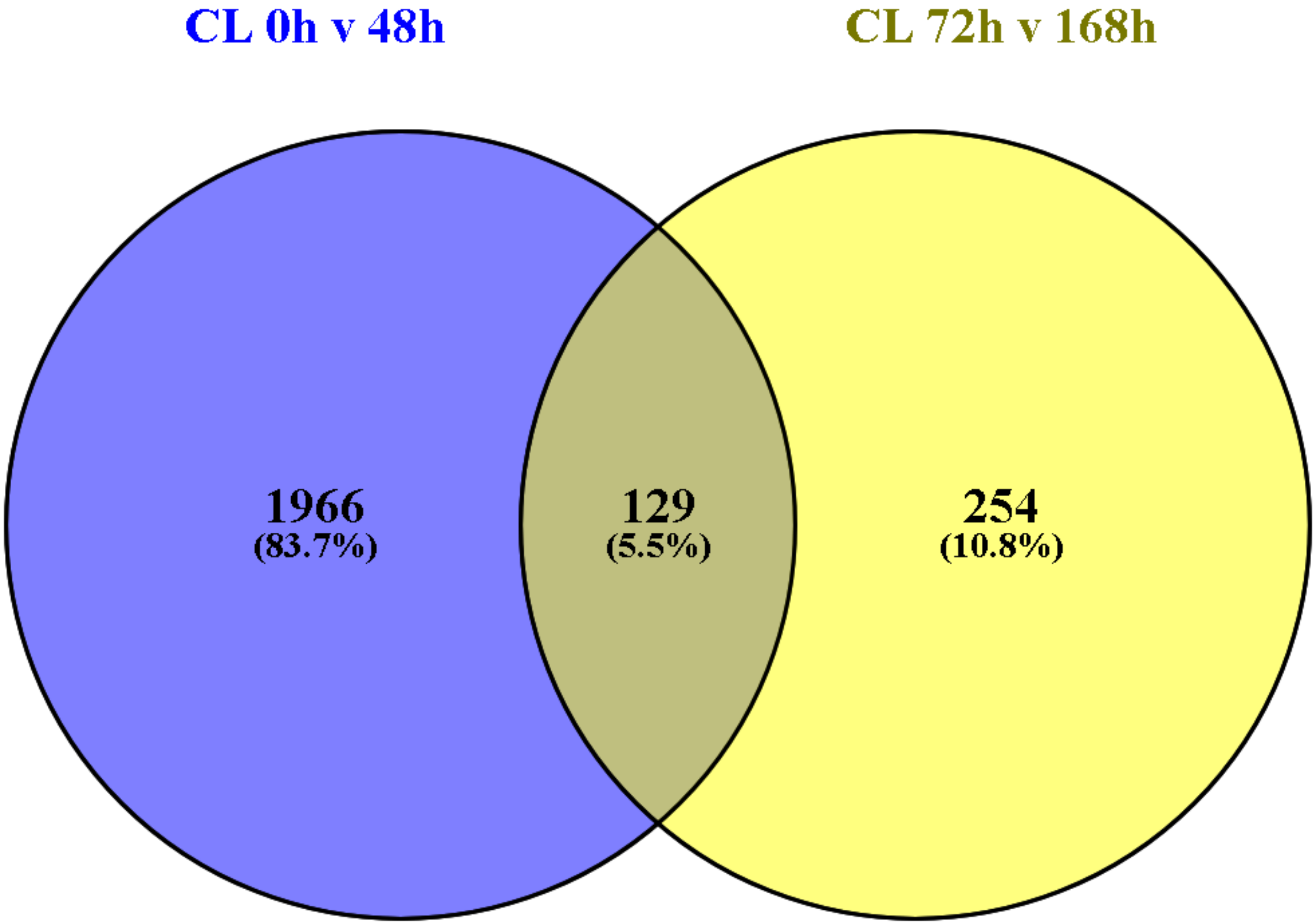

Supplementary Figure 8

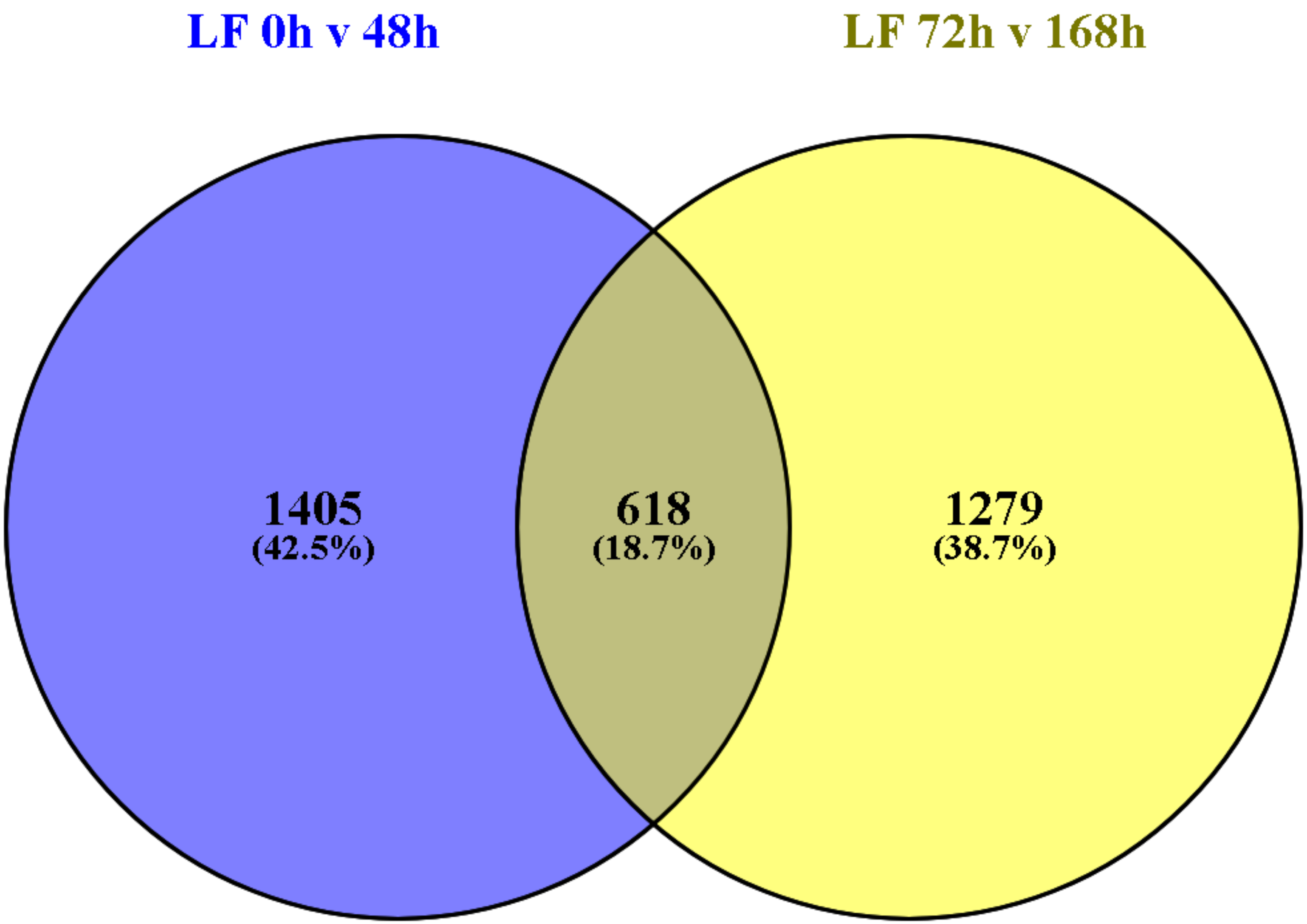

Supplementary Figure 9

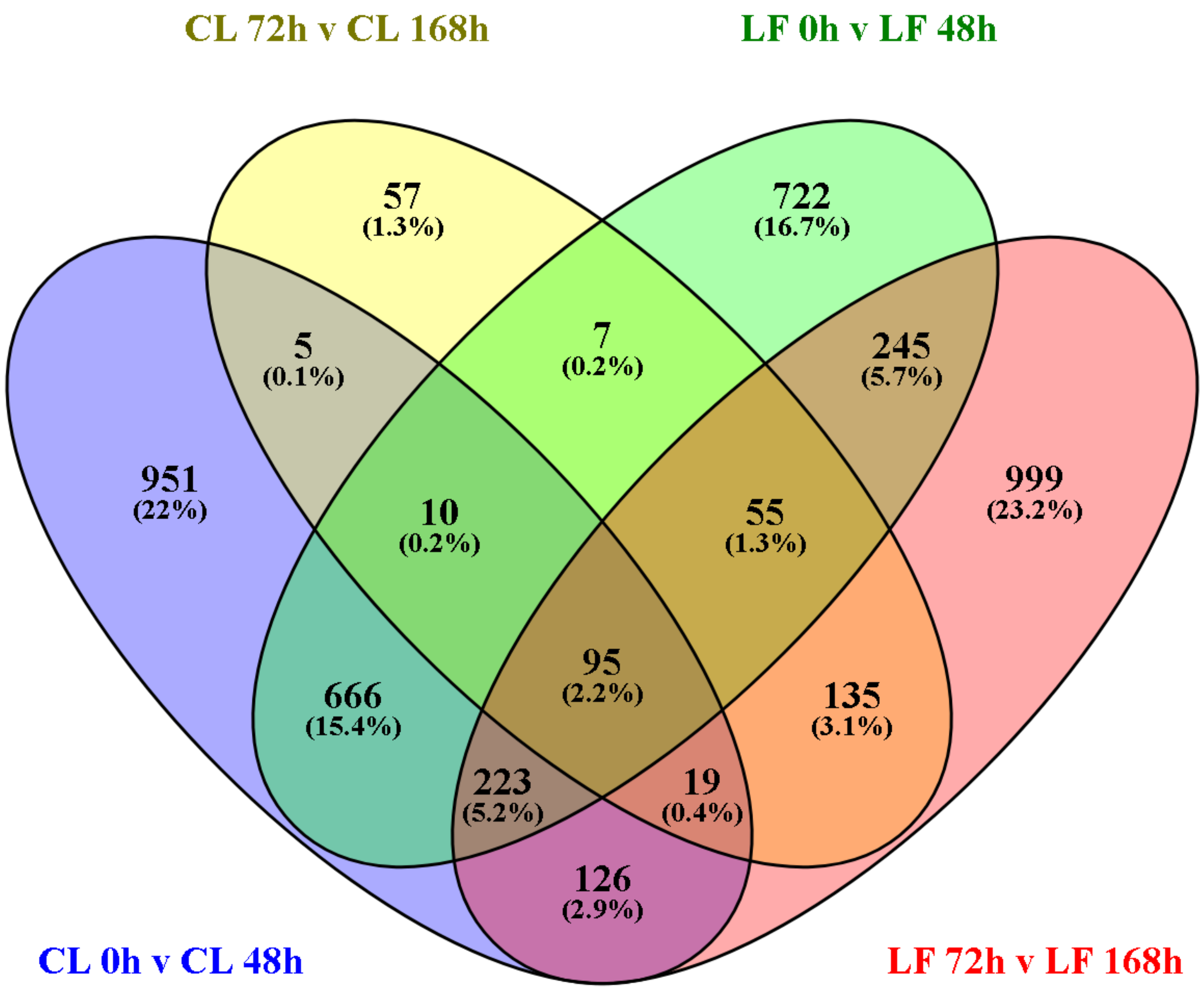

Supplementary Figure 10
Beef CL 48h v CL 0h
Fibrogenesis and transmigration of cells network regulators upregulated in 48hour

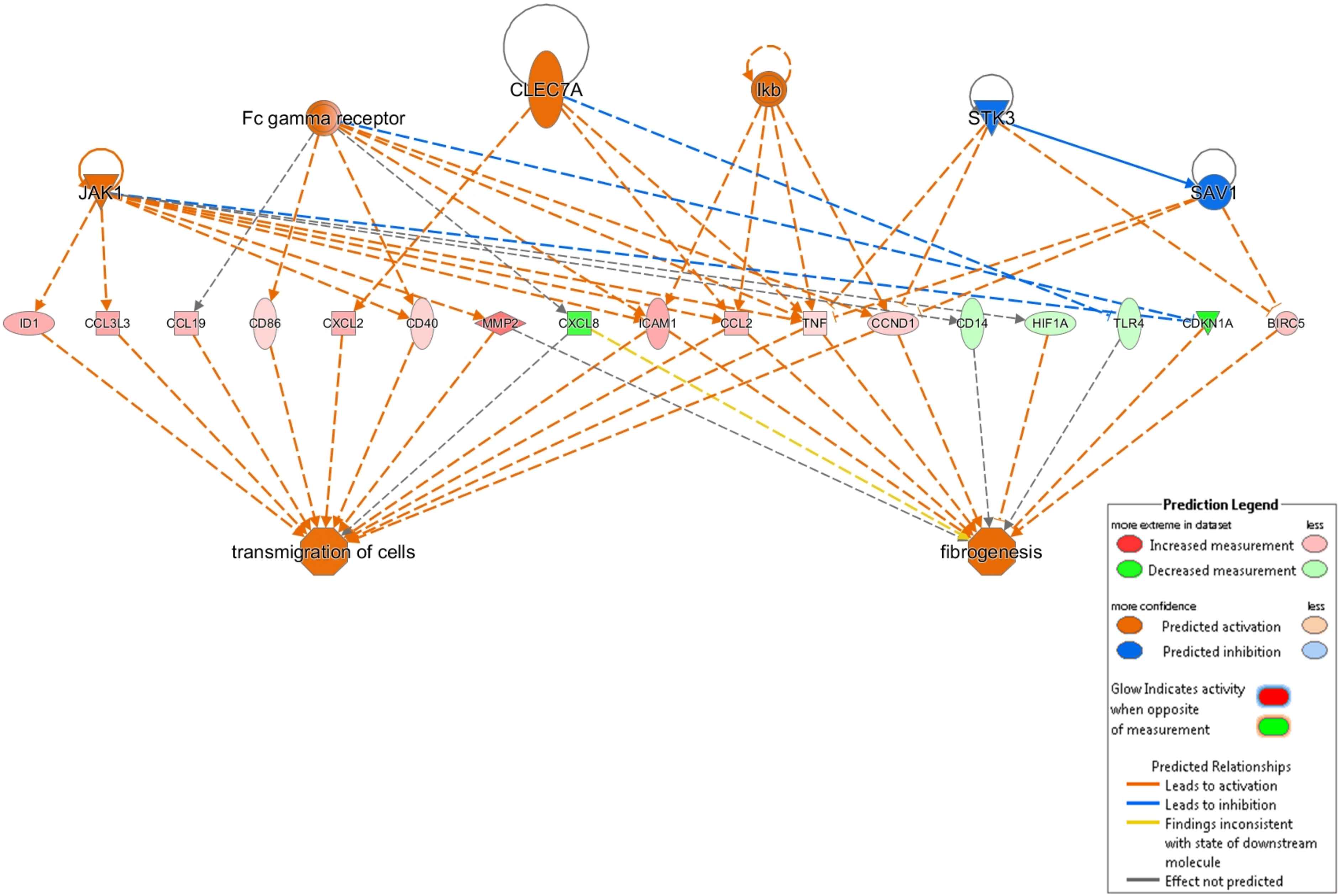

Supplementary Figure 11
Beef CL168h v CL 72h
Immune system overall responses inhibited at 168 hours

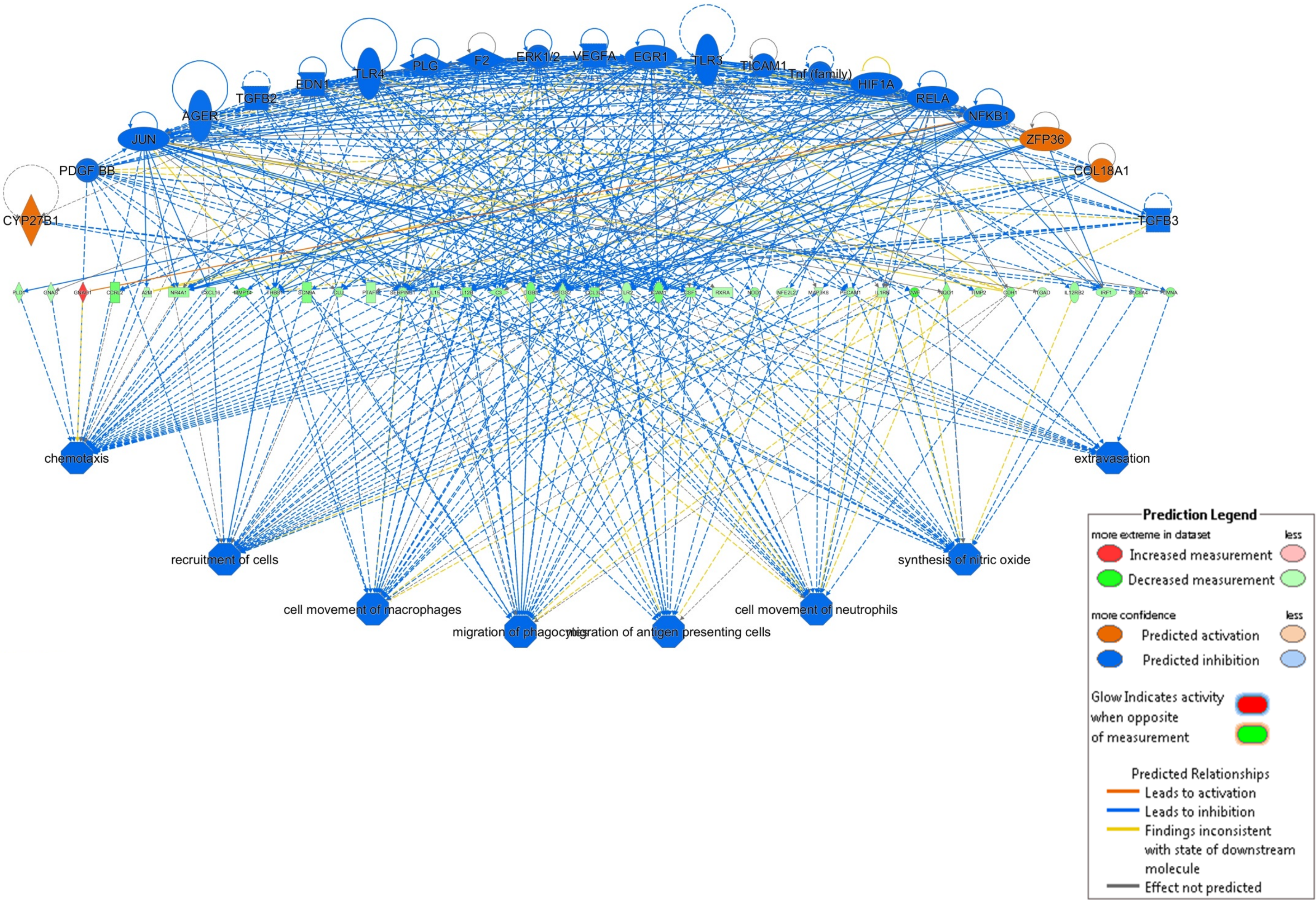

Supplementary Figure 12
Beef LF 168h v Beef LF 72h
Hematological System Development and Function, Immune Cell Trafficking, Inflammatory Response
Network 14

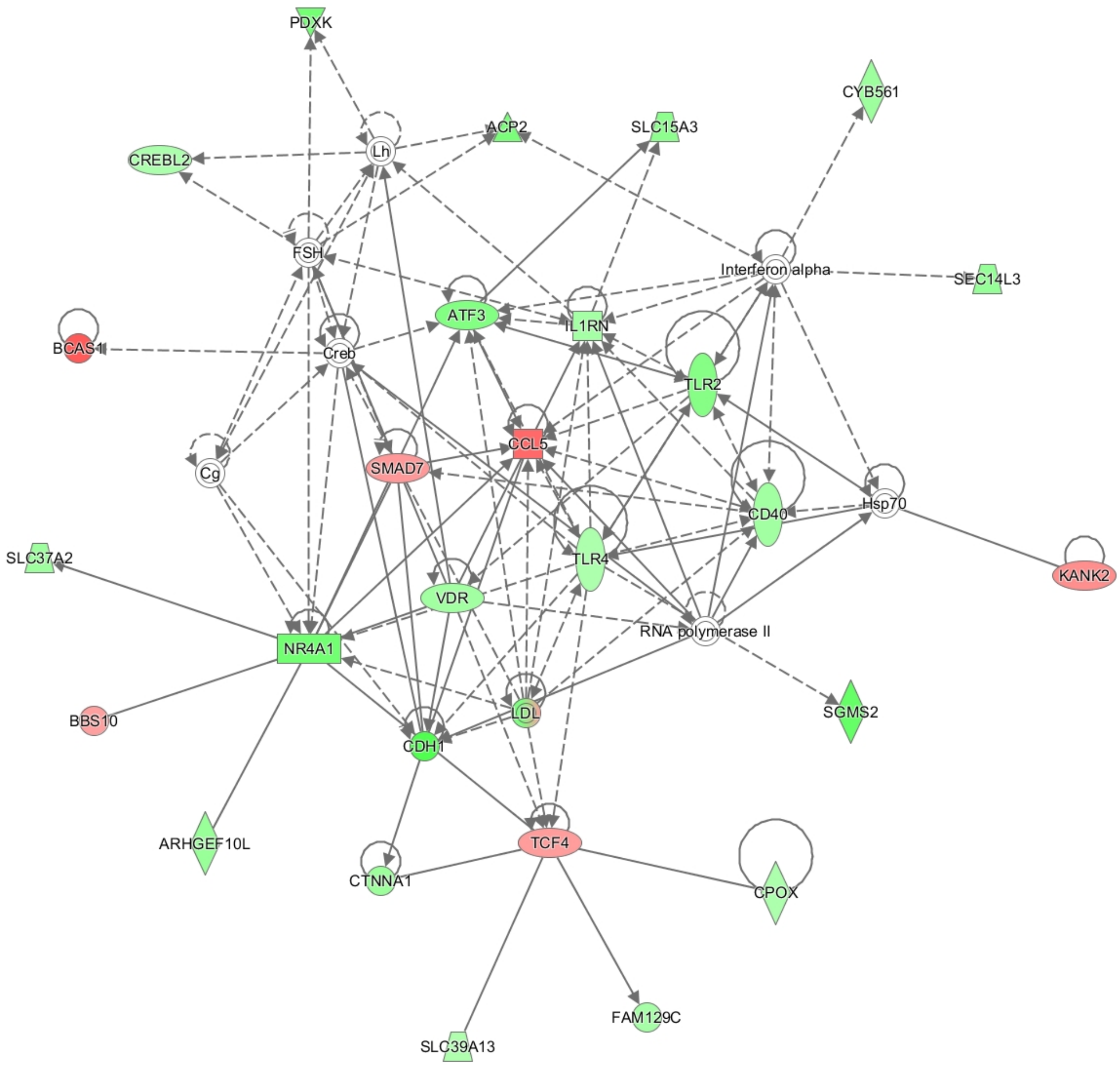

Supplementary Figure 13
Beef CL 48 v LF 48
Inhibition of CD40l in CL48- The cytokine that binds to CD40

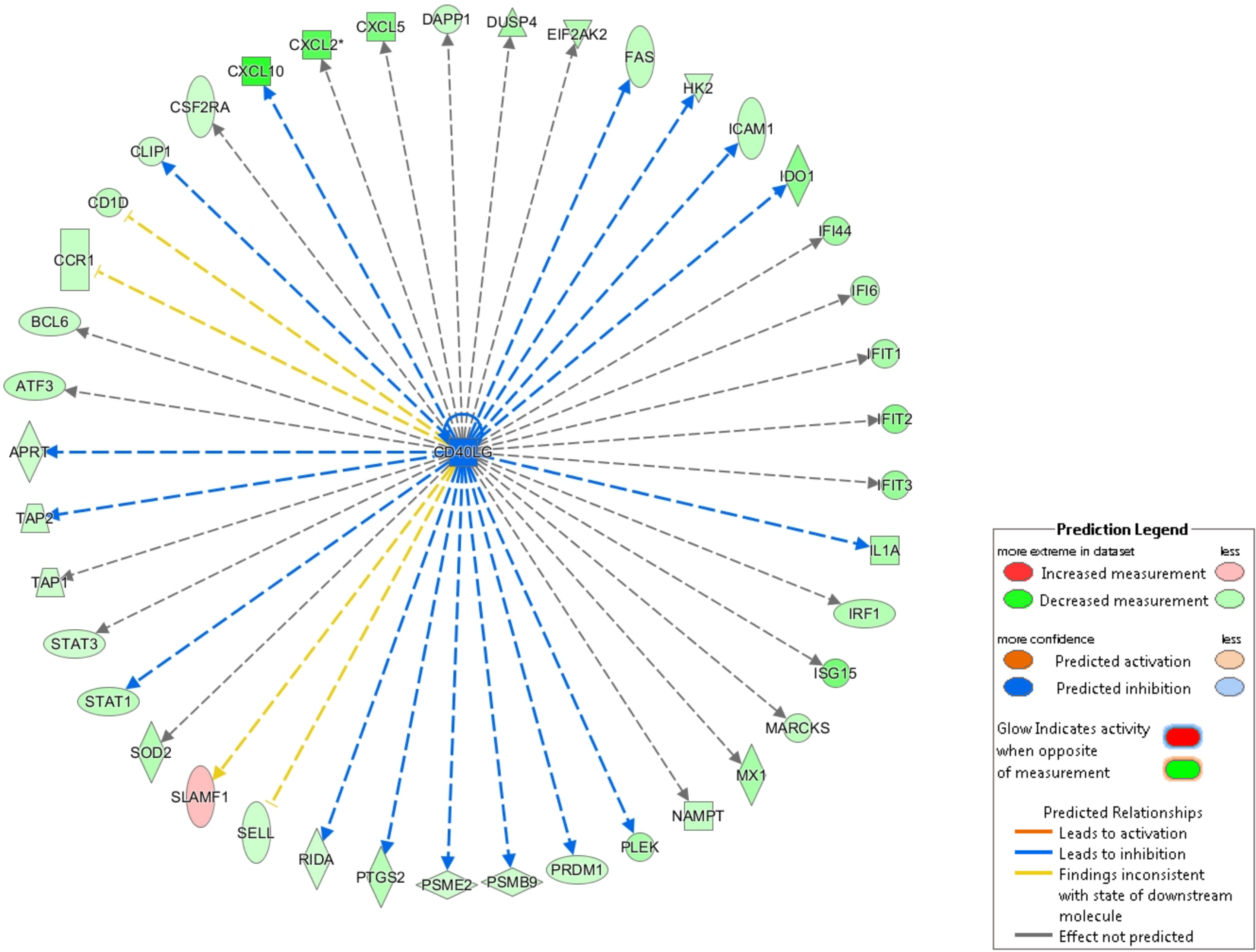

Supplementary Table 1

Calf milk replacer and concentrate composition

DM (dry matter); CP (Crude protein)

**Chemical composition**	**Calf diet**		
	Milk replacer	Concentrate	Straw
Oven DM[1] (g/kg fresh)	972	916	882
Composition of DM (g/kg)			
Nitrogen	36.6	29.6	5.7
NDF[2]	-	347.5	869.3
ADF[3]	-	175.9	530.5
Ash	75.1	75.4	47.3
Ether extract	201.9	42.4	13.9
CP[2] (%)	22.8	18.5	3.6
